# Specificity Protein 1 Regulates Gene Expression Related to Fatty Acid Metabolism in Goat Mammary Epithelial Cells

**DOI:** 10.3390/ijms16011806

**Published:** 2015-01-14

**Authors:** Jiangjiang Zhu, Yuting Sun, Jun Luo, Min Wu, Jianhua Li, Yanhong Cao

**Affiliations:** 1 Shaanxi Key Laboratory of Molecular Biology for Agriculture, College of Animal Science and Technology, Northwest A&F University, Yangling 712100, China; E-Mails: zhujiang4656@hotmail.com (J.Z.); yutingsun2008@126.com (Y.S.); minwu1229@163.com (M.W.); lijianhua991@gmail.com (J.L.); 2 Guangxi Institute of Animal Husbandry, Guangxi Zhuang Autonomous Region, Nanning 530000, China; E-Mail: caoyh610@163.com

**Keywords:** dairy goat, mammary epithelial cells, specificity protein 1, fatty acid metabolism

## Abstract

Specificity protein 1 (SP1) is a ubiquitous transcription factor that plays an important role in controlling gene expression. Although important in mediating the function of various hormones, the role of SP1 in regulating milk fat formation remains unknown. To investigate the sequence and expression information, as well as its role in modulating lipid metabolism, we cloned *SP1* gene from mammary gland of *Xinong Saanen* dairy goat. The full-length cDNA of the *SP1* gene is 4376 bp including 103 bp of 5'UTR, 2358 bp of ORF (HM_236311) and 1915 bp of 3'UTR, which is predicted to encode a 786 amino acids polypeptide. Phylogenetic tree analysis showed that goat *SP1* has the closest relationship with sheep, followed by bovines (bos taurus, odobenus and ceratotherium), pig, primates (pongo, gorilla, macaca and papio) and murine (rattus and mus), while the furthest relationship was with canis and otolemur. Expression was predominant in the lungs, small intestine, muscle, spleen, mammary gland and subcutaneous fat. There were no significant expression level differences between the mammary gland tissues collected at lactation and dry-off period. Overexpression of *SP1* in goat mammary epithelial cells (GMECs) led to higher mRNA expression level of peroxisome proliferator-activated receptor-γ (*PPARγ*) and lower liver X receptor α (*LXR*α) mRNA level, both of which were crucial in regulating fatty acid metabolism, and correspondingly altered the expression of their downstream genes in GMECs. These results were further enhanced by the silencing of *SP1*. These findings suggest that *SP1* may play an important role in fatty acid metabolism.

## 1. Introduction

The importance of goats as essential food in meat and dairy industries has been discussed and documented [1]. High contents of beneficial fatty acids endow goat milk with a high nutritional value, which also prevents some human metabolic disorders [1,2]. Although several genes including peroxisome proliferator-activated receptor-γ (*PPARγ*) [3], sterol regulatory element binding proteins 1 (*SREBP*1) [4] and liver X receptor α (*LXRα*) [5], have been reported to be crucial in regulating milk fat metabolism, the regulatory mechanisms of lipid metabolism remain unclear.

Specificity protein 1 (SP1), a member of Sp/Kruppel super family [6], is important for transcription of many cellular and viral genes that contain GC boxes in their promoters [7], particularly to those genes related to biosynthesis and metabolism of nucleic acids (e.g., thymidylate synthase, adenine deaminase and DNA polymerase), cell cycle and proliferation [8]. It regulates the expression of genes by binding to the GC-rich domains (GGGGCGGGG/GGTGTGGG) in gene promoters to enhance transcription [8,9]. In the absence of a TATA-element, SP1 also facilitates a sequential recruitment of transcription factor IID (TFIID) and RNA polymerase II [10,11,12]. Based on this evidence, SP1 is well known as a basal transcription factor [6].

Increasing reports are revealing that SP1 may be involved in lipid metabolism regulation. SP1 has been shown to be one of the major transcription activators of FASN [13], the crucial enzyme for *de novo* fatty acid synthesis. In breast cancer cells the silencing of *SP1* expressed significantly lower levels of FASN and SREBP-1c mRNA [14]. Similarly, SP1 overexpression enhanced the expression of human FASN in the presence of SREBP-1c [13]. To the contrary, decreased FASN-promoter activity was observed without the help of SREBP1 in rat hepatocytes [15]. It is a fact that, 34% of SREBP1 targets are occupied by SP1, which is partly explained by synergistic action, [16] such as, ATP-citratelyase (ACLY) and acetyl-CoA carboxylase (ACACA) both of which are downstream of SREBP1 [17] and contain SP1 binding sites in the promoters [18,19]. Besides, SP1 functions as a co-factor of signal transducer and activator of transcription 3 (Stat3) [7], activates the promoter of acyl-coenzyme-A oxidase (ACOX), the rate-limiting enzyme in peroxisomal beta-oxidation of fatty acids, in synergy with peroxisome proliferator-activated receptor (PPARγ) or retinoid-X receptor (RXR) [20]. Moreover, SP1 and hypoxia-induced factor 1 (HIF1) were also reported to co-regulate the expression of ATP-binding cassette A1 (ABCA1), which is one of the major regulators in mediating cellular efflux of phospholipids and cholesterol [21]. Overall, these literatures suggest that SP1 may also play an important role in regulating expression of genes involved in lipid metabolism. Despite the importance of SP1, lack of sequence and expression information hindered functional research in dairy goats.

In the present study, we cloned the full-length cDNA of the *SP1* gene from mammary gland of *Xinong Saanen* dairy goats and analyzed the sequence by bioinformatics. A tissue extensive expression distribution was identified. The effect of *SP1* expression levels on the expression of *PPARγ*, *SREBP1* and *LXRα* were investigated in goat mammary epithelial cells (GMECs) with adenovirus-mediated overexpression and siRNA-mediated RNA interference respectively. These basal data may provide original sequence and expression information of the *SP1* gene and is also better for our understanding about the regulatory role of *SP1* in milk fat formation in mammary gland of dairy goats.

## 2. Results

### 2.1. Characterization of SP1 cDNA from Goat Mammary Gland

The full-length cDNA of *SP1* gene is 4376 bp, includes 103 bp of 5'UTR, 2358 bp of ORF (HM_236311) and 1915 bp of 3'UTR, predicted to encode a 786 amino acids polypeptide. An ATG initiation codon is located at 104 nt, and a TAA stop codon is situated at 2462 nt. The sequence also contains one AACAAA motifs, which represent putative polyadenylation signals (nt3893–3898) (Figure S1). The coding sequence (CDS) of goat *SP1* shares 99.6%, 94.7% and 91.9% similarity with bovine (NM_001078027.1), human (NM_138473.2), and rattus (NM_013672.2) respectively. Correspondingly, the similarity of the amino acid sequence is 99.3%, 96.9% and 95%, respectively. The similarity of 5'UTR is 100%, 97.6% and 96.8%, and that of the 3'UTR is 97%, 86% and 86%, with previously indicated species, respectively (Figure S2).

The SP1 protein has a calculated molecular weight of 80,840.1-Da and isoelectric point (PI) of 7.23. The most abundant amino acid is serine (Ser), which contains up to 100 amino acids, accounts for 12.7% of total amino acids. It contains 41 negatively charged residues (Asp and Glu) and 41 positively charged residues (Arg and Lys). The estimated half-life of the protein is 30 h in mammalian reticulocytes *in vitro*, 20 h in yeast *in vivo* and 10 h in *Escherichia coli*, respectively. This protein is computed to be unstable with 52.26 of instability index (II) and 68.80 of aliphatic index. The grand average of hydropathicity (GRAVY) is −0.440, max at 1.978 and min at −3.2 (Figure S1).

Before the *N*-terminus Met1…Gly29 residues, is a signal peptide with no transmembrane helix. An acetylation site is predicted at Ser2, and three zinc fingers are predicted in Cys529…His551, Cys559…His581 and Cys589…His709, respectively. The SP1 protein contains 45 serine phosphorylation sites, 9 threonine phosphorylation sites, 3 tyrosine phosphorylation sites and 11 glycosylation sites. In addition, the SP1 protein contains 2 GLN rich regions (Gln156…Gln213 and Gln352…Gln481) and 1 SRE-rich region (Ser271…Ser328) (Figure S1).

**Figure 1:**
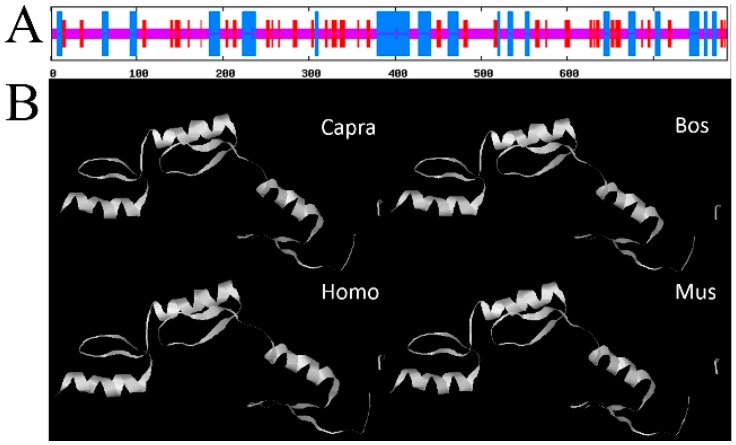
Structure prediction of SP1 proteins. (**A**) The secondary structure of SP1 protein. Blue color represents alpha helixes, red color represents beta bridges, and purple represents random coils; (**B**) The tertiary structure of SP1 protein in various species.

We also investigated the secondary structure and compared the tertiary structure of SP1 protein of capra hircus, bovine, human, and mouse. The protein of SP1 contains 3 alpha helices-rich regions (Figure 1A). The spatial structure of SP1 protein shares a high similarity among different species (Figure 1B).

### 2.2. Phylogenetic Analysis Based on SP1 Amino Acid Sequences

**Figure 2:**
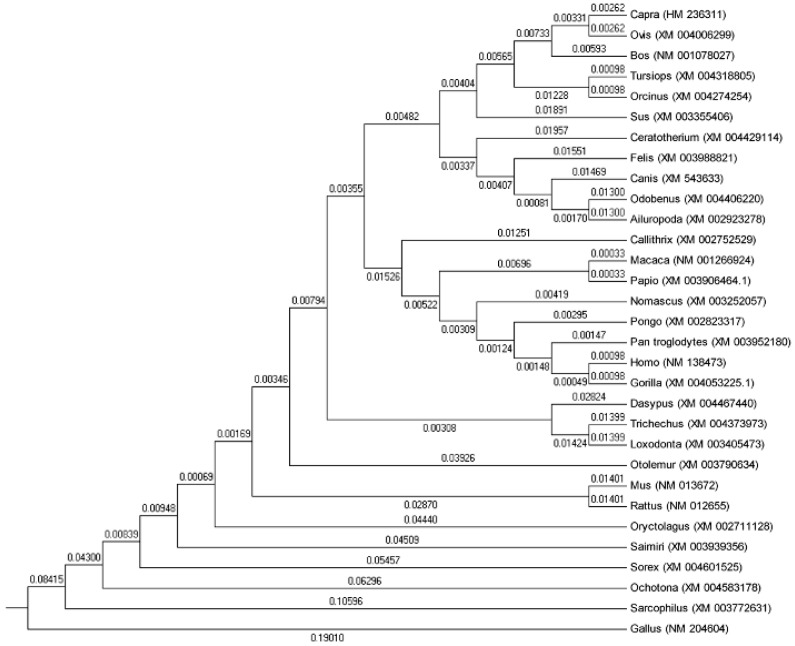
Phylogenetic tree based on *SP1* gene sequences of 31 representative animals made with MEGA 5 software (S. Kumar, Tempe, AZ, USA) using Neighbor-Joining (NJ) method.

To investigate potential evolutionary processes of the *SP1* gene among various species, a neighbor-joining phylogenetic tree was constructed, based on sequences of 31 representative animals. The result shows that goat SP1 has the closest relationship with sheep, followed by bovines (including bos taurus, odobenus and cerato therium), pig, primates (pongo, gorilla, macaca and papio) and murine (rattus and mus). Canis and otolemur show the greatest distance from capra hircus (Figure 2).

### 2.3. Analysis of the Expression Profile of Goat SP1

**Figure 3:**
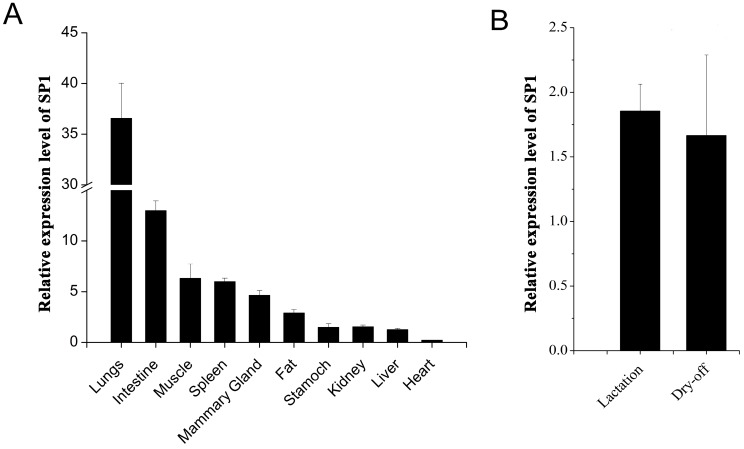
The mRNA expression profiles of *SP1*. (**A**) mRNA expression of *SP1* in various tissues in dairy goats; (**B**) Expression level of *SP1* between lactation and dry-off periods.

In order to enhance understanding the role of *SP1* in various tissues of *Xinong Saanen* dairy goat, we investigated the mRNA expression profiles of *SP1*. *SP1* was expressed at highest levels in lungs and small intestine, followed by muscle, spleen, mammary gland and subcutaneous fat tissues. Stomach, kidney and liver showed similar *SP1* expression levels. Heart had the lowest expression mRNA level among the tissues examined (Figure 3A).

To investigate whether the expression was affected by different lactation stages, we assayed *SP1* mRNA level from the tissue samples in the lactation and dry-off periods. However, we did not find any significant expression differences between the two periods, though a tendency of higher expression was observed from lactation samples (Figure 3B).

### 2.4. SP1 Is Involved in Lipid Metabolism Regulation

**Figure 4:**
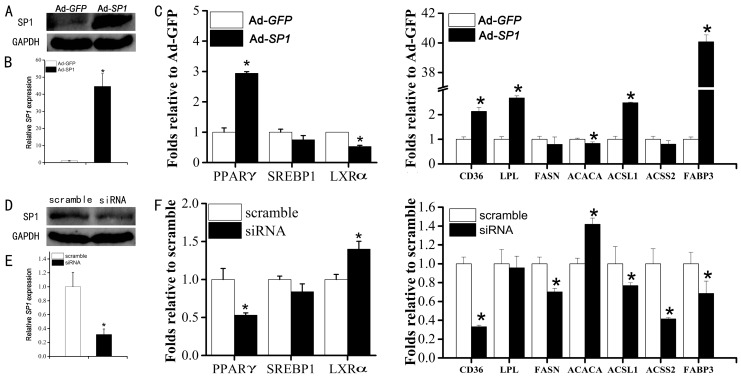
Effect of expression alteration of SP1 on the expression of genes relative to lipid metabolism. (**A**) Ad-*SP1* overexpressed the expression level of SP1 protein; (**B**) Ad-*SP1* overexpressed the mRNA expression level of the *SP1* gene; (**C**) Overexpression of *SP1* altered the mRNA expression level of genes related to lipid metabolism in goat mammary epithelial cells (GMECs); (**D**) siRNA decreased the protein expression of SP1; (**E**) siRNA decreased the mRNA expression of *SP1*; and (**F**) The silencing of *SP1* affected the mRNA expression of genes associated with lipid metabolism. Columns, average of 3 repeats; bars, SD; *, *p* < 0.05

After treatment with adenovirus of Ad-*SP1* for 72 h, both the protein (Figure 4A) and mRNA expression (Figure 4B) of SP1 were significantly increased. Accompanying the overexpression of the *SP1* gene, an obvious increase was observed in *PPARγ* expression, while *LXRα* was decreased dramatically by adenovirus treatment (Figure 4C). Corresponding to the result of *SP1* overexpression, silencing of *SP1* (Figure 4D,E) decreased the expression of *PPARγ* and increased the expression of *LXRα* significantly (Figure 4F). However, *SREBP1* remained unaffected after adenovirus or siRNA treatments, though a slight decrease was observed (Figure 4C,F).

Relative to the control (Scramble, negative siRNA), silencing of *SP1* markedly decreased the expression of CD36 molecule (*CD36*), acyl-CoA synthetase long-chain family member 1 (*ACSL1*) and heart type fatty acid bind protein (*FABP3*). All of these results were enhanced by the increased expression of these genes in Ad-*SP1* treatment. The expression of *ACACA* was increased by the loss of SP1 and decreased in adenovirus treatment. The expression of lipoprotein lipase (*LPL*) was upregulated with the overexpression of SP1, but not changed significantly by the reduction of SP1. Conversely, while *FASN* and cytosolic acetyl-CoA synthase (*ACSS2*) were not affected by Ad-*SP1* treatment relative to the control (Ad-*GFP*), the loss of SP1 reduced their mRNA content markedly.

## 3. Discussion

SP1-like proteins and Krüppel-like factors (KLFs) are highly related zinc-finger proteins that are important components of the eukaryotic cellular transcriptional machinery. By regulating the expression of a large number of genes containing GC-rich promoters, SP1-like/KLF transcription regulators virtually take part in all facets of cellular function, including cell proliferation, apoptosis, differentiation, and neoplastic transformation [22]. As a member of the super-family, although SP1 was mainly regarded as a basal transcription factor [6], increasing reports have indicated that SP1 is involved in the regulation of lipid metabolism, enabled by the zinc finger structures [22], which comprise a DNA-binding domain that also serves as a nuclear localization signal (NLS) [23]. Based on these reports, SP1 is assumed to play an important role in regulating milk fat formation. In this study, we firstly cloned the full-length cDNA of *SP1* (HM_236311) from mammary gland of *Xinong Saanen* dairy goat. The protein, which is composed of 786 amino acid residues, contains three zinc fingers in the carboxyl terminal, similar to the other members of the super-family [22]. In addition, 2 GLN rich regions (Gln156…Gln213 and Gln352…Gln481) and an Ser/Thr rich regions, represented by SRE-rich regions, were predicted in the present study, both of which were reported to be shared among SP1-like proteins (SP1, SP2, SP3 and SP4), and predicted to be crucial for their function [22]. Thus, it is proposed that goat SP1 may share a similar function in regulating gene expression with the other members of SP family.

SP1 phosphorylation has been tied to the functional changes of DNA binding and promoter activation, which leads to expression of the target genes [24]. SP1 phosphorylation induced by oleic acid treatment led to an enhanced association with other transcription factors to synergistically increase HIV-LTR promoter activity. Further, SP1 can be phosphorylated at various sites or combination of sites, leading to a wide range of changes in SP1 function [24]. Different kinases and phosphatases targeted selected motifs to alter SP1 phosphorylation in a cell-type-specific manner [25]. The phosphorylation is predominantly on serine residues with less than 5% on threonine and none on tyrosine residues [24]. In humans the phosphorylation sites of Ser59, Ser131, Thr453, Thr579 (located in the second zinc finger) and Thr739 have been identified to be important for regulating DNA binding and gene expression. Here, we predicted 58 potential phosphorylation sites (45 serine phosphorylation sites, 10 threonine phosphorylation sites and 3 tyrosine phosphorylation sites) in the SP1 protein, including Ser59, Thr453, Thr579 and Thr739, and also found that they share a high similarity among different species (as shown in Figure S2). This indicates that goat SP1 may also play an important role in DNA promoter binding and gene expression.

In addition to phosphorylation, SP1 also contains multiple potential *O*-glycosylation sites that may be modified by *N*-acetylglucosamine residues, which has been suggested to determine the stability of cellular SP1 [26]. Reduced *O*-glycosylation of SP1 was identified to be associated with increased proteasome susceptibility in HeLa cells [26]. Additionally, the glycosylation state of SP1 is highly correlated with its ability of gene transactivation [27]. For example, *O*-GlcNAc modification is able to inhibit the interaction between SP1 peptide and TATA-binding protein-associated factor (TAF110) and holo-Sp1 in HeLa cells [28]. Corresponding to previous studies, we found 11 potential glycosylation sites in the SP1 protein, with 4 serine glycosylation sites and 7 threonine glycosylation sites, suggesting that goat SP1 may play an extensive role in milk fat metabolism regulation.

The results of tissue expression showed that *SP1* is mainly expressed in the tissues related to strong lipid metabolism in dairy goats, including lungs, small intestine, subcutaneous fat, mammary gland and so on, suggesting an important role in regulating lipid metabolism. However, there was no significant difference between lactation period and dry-off period. Besides, the activity of SP1 was mainly regulated by the phosphorylation level induced by stimulation from outside. This may explain the stable state of the mRNA level of *SP1*.

Despite the importance of SP1 in human [14] and rat [6], rare studies reported on SP1 regulated lipogenesis in ruminants’ mammary gland. PPARγ and SREBP1 are central for milk fat synthesis regulation, and highlight a pivotal role in regulating gene expression associated with lipid metabolism [29]. LXRα is considered as a critical transcription factor for the activation of a number of genes [5]. PPARγ and SP1 were co-localized in the nuclei of the bovine embryo binucleated cells on Day 25 [30], and interacted with SP1 to regulate the expression of the VEGF receptor 2 [31]. The SREBP-1c promoter was accelerated by ectopic expression of SP1 and insulin further enhanced the transactivation potential of SP1. Silencing of *SP1* reduced both basal and insulin-induced activation of the SREBP-1c promoter. We also found that Sp1 interacted with both SREBP-1c and LXR proteins and insulin promoted these interactions [32]. Thus, it is speculated that *SP1* may be involved in milk fat formation regulation via the control of these transcription factors. Here, for verification, overexpression of *SP1* significantly increased the expression of *PPARγ* and *LXRα*. These results were also validated by the result of siRNA treatment. However, the expression of *SREBP1* remains unaffected by either overexpression or knockdown of *SP1*, indicating its insignificant role in responses to *SP1* alteration. Further investigation concerning the silencing of *PPARγ* and *LXRα* may be helpful for our understanding of the mechanisms of *SP1* involvement in lipid metabolism regulation.

For verification of results, we detected the mRNA level of the target genes of *PPARγ* and *LXRα*. The corresponding expression change of *CD36*, *LPL ACSL1* and *FABP3*, all of which were reported to be downstream of *PPARγ* [3,29], and induced by the alteration of *PPARγ*, confirmed that *SP1* may regulate fatty acid uptake (CD36 and LPL) and long-chain fatty acids transportation (ACSL1 and FABP3) via the control of expression of *PPARγ*. The mutation of the GC region of the bovine *FASN* gene promoter reduced the binding of SP1 and also decreased the promoter activity [33]. ACACA and ACSS2 have been shown to be regulated by both LXRα and SREBP1 [34]. The alteration of ACACA in the present study was consistent with the research in bovine, in which the promoter of the ACACA gene harbors nine copies of these GC boxes known as the binding sites for the transcription factor SP1 [35]. Being considered to be the main pathway, LXRα can also alter lipid metabolism through other means [36]. In the present study, the expression of *ACACA* and *ACSS2* were changed followed the alteration of *LXRα* but not *SREBP1*, suggesting that *LXRα* may play an important role in mediating the function of *SP1* in GMECs. This hypothesis was further enhanced by the stable expression of *FASN*, the main target gene of *SREBP1*, with altered the expression of *SP1*.

## 4. Experimental Section

### 4.1. Animals and Samples Collection

Six *Xinong Saanen* dairy goats (four years old, third parity) were selected and divided into two groups of three to conduct the main project (Experimental Farm of the Northwest A&F University, Yangling, China). For one group, all three goats were slaughtered during the lactation period (100 days), tissue samples (lungs, small intestine, muscle, spleen, mammary gland, subcutaneous fat, stomach, kidney, liver and heart) were rapidly collected, and rinsed with DEPC (Sigma, St. Louis, MO, USA) sterile water, and were then used for tissue expression determination of the *SP1* gene [37,38,39,40]. Dry-off period was presumed from 300 days after calving to the next calving. Here, for the comparison with the expression of *SP1* in the lactation period, goats in the other group were slaughtered during the dry period (310 days after calving with pregnant). Mammary gland tissue samples were collected from each goat under sterile procedures to investigate the expression of *SP1* in different lactation periods [41,42,43]. All samples were stored in liquid nitrogen. The Animal Care and Use Committee of the Northwest A&F University approved all procedures and experiments.

### 4.2. Total RNA Extraction and Real-Time Quantitative PCR (RT-qPCR)

Total RNA were extracted from approximately 100 mg tissue of slaughtered goats (listed above) using trizol reagent (Invitrogen, Shanghai, China) and treated with RNase-free DNase (CWBIO, Beijing, China) for removing DNA contamination. The concentration of RNA was analyzed with the NanoDrop 2000 (NanoDrop Technologies, Wilmington, DE, USA). The purity of RNA (A260/A280) for all samples was above 2.0. The RNA quality was verified by agarose gel electrophoresis analysis of 28S and 18S rRNA stained with ethidium bromide. The first-strand cDNA of various tissues were synthesized from 500 ng of purified total RNA using the PrimeScript™ RT kit (for perfect Real-time) (Takara, Otsu, Japan) according to the manufacturer’s instructions. SYBR^®^ Premix Ex Taq™ II (Takara, Japan) was used for measuring the relative expression level of *SP1* in various tissues by RT-qPCR fluorescent technology according to the manufacturer’s manual on a CFX96 Real-Time PCR Detection System (Bio-Rad, Hercules, CA, USA). PCR amplification was carried out at 95 °C for 4 min, followed by 40 cycles of 95 °C for 15 s, 60 °C for 30 s and 72 °C for 30 s. The presence of a single PCR product was verified by the dissociation protocol using incremental temperatures to 95 °C for 15 s plus 65 °C for 5 s. Ubiquitously expressed transcript (UXT), mitochondrial ribosomal protein L39 (MRPL39) [44] and glyceraldehyde-3-phosphate dehydrogenase (GAPDH) [41,45] was used as an endogenous reference for normalization of targeted mRNA profiles (Table S1).

### 4.3. cDNA Cloning of SP1 from Dairy Goat Mammary Gland

The primers for *SP1* cloning, as described in Table S2, were designed based on the multiple alignment of the conserved sequence of sheep (ovis, XM_004006299), bovine (Bos taurus, NM_001078027.1), human (Homo sapiens, NM_003109.1) and rattus (Rattus norvegicus, NM_013672.2). Briefly, PCRs for open reading frame (ORF) cloning were performed with an initial denaturation step at 95 °C for 5 min, 25 cycles at 94 °C for 30 s, with 1 °C/cycle gradient temperature annealing from 50 °C for 30 s and extension at 72 °C for 2.5 min. This was followed by a 10 min extension at 72 °C. The cDNA cloning of the 5'- and 3'-UTR were performed by nested PCR according to the manufacture’s protocol of 5' RACE system Ver.2.0 kit (Invitrogen, USA) and 3'-full RACE core set Ver.2.0 kit (Clontech, Takara Bio Group, Otsu, Japan), respectively. All the PCR fragments were cloned into pMD^®^ 19-T (Takara, Otsu, Japan) vectors and sequenced by Invitrogen (Carlsbad, CA, USA).

### 4.4. Sequence Analyses

The core fragment, 3' end and 5' end sequences were assembled using the SeqMan II software in the DNAStar Package (DNAStar Inc., Madison, WI, USA) to obtain full-length cDNA of *SP1*. The cross-species similarities of both nucleotide and putative amino acid sequences were then calculated for homology on ClustalW (http://www.ebi.ac.uk/Tools/clustalw2/index.html). Exon information was predicted by the NCBI online tool (http://www.ncbi.nlm.nih.gov/sutils/splign/splign.cgi?textpage=online&level=form). Protein sequence, chylomicron (MW) and isoelectric point values (PI) were analyzed by the protparam program of EXpASy (http://www.expasy.org/cgi-bin/protparam). ProtScale (http://us.expasy.org/cgi-bin/protscale.pl) was used for hydrophobicity structure prediction, TMHMM (http://www.cbs.dtu.dk/services/TMHMM/) was used for trans-membrane structure prediction, SignalP (http://www.cbs.dtu.dk/services/SignalP/) was used for signal peptide sequence prediction. Prosite program of ExPASy (http://www.expasy.org/cgi-bin/prosite/ScanView.cgi?scanfile=294556012219.scan.gz) was used for SP1 protein domains analyses. CBS netOGlyc 3.1 was used for glycosylation sites prediction (http://www.cbs.dtu.dk/services/NetOGlyc/). CBS Netphos 2.0 was used for phosphorylation sites prediction (http://www.cbs.dtu.dk/services/NetPhos/). CBS NetAcet 1.0 was used for acetylation site prediction (http://www.cbs.dtu.dk/services/NetAcet/). Phyre2 was used for protein tertiary structure prediction (http://www.sbg.bio.ic.ac.uk/~phyre2/html/page.cgi?id=index).

### 4.5. siRNA Synthesis and Adenovirus Generation

siRNA targeting SP1 and control siRNA (scramble) were designed and synthesized according to the sequence observed previously (Invitrogen, USA). siRNA sequence was as following, Sense: 5'-GCCAAUAGCUACUCAACAATT-3', anti-sense: 5'-UUGUUGAGUAGCUAUUGGCTT-3'. Control sequence was as following, sense: 5'-UUCUCCGAACGUGUCACGUTT-3', anti-sense: 5'-ACGUGACACGUUCGGAGAATT-3'. siRNA were then transduced following the reverse transfection method described in the manual of Lipofectamine™ RNAiMAX (Invitrogen, Carlsbad, CA, USA). Adenovirus contain *GFP* sequence (Ad-*GFP*) and *SP1* sequence (Ad-*SP1*) were generated followed the protocol described previously [45]. After proliferation, high titer adenovirus were used for transfecting GMECs.

### 4.6. Cell Culture and Treatment

HEK 293 cells were cultured in Dulbecco’s modified Eagle medium (DMEM) (Hyclone, Beijing, China) compensated with 10% fetal bovine serum (FBS) (Hyclone, Beijing, China). Mammary gland samples from three healthy dairy goats at peak lactation were collected after slaughter. GMECs purification and primary culture was according to our previous study [46]. Briefly, after slaughtering, the mammary tissues were collected and trimmed of visible fat and connective tissues and washed with D-Hank’s solution several times. Acini were then selected and minced into about 1 mm^3^ cubes after being rinsed again with D-Hank’s solution and kept up for 10 min at room temperature. The smaller pieces of tissues were put onto the dishes for culture. The new generated cells were then purified according to trypsin digestion method for five passages, which was presumed to remove other cells types, mainly including fibroblasts. The purified GMECs were cultured as in our previous study [47]. Briefly, GMECs were cultured in DMEM/F-12 (Hyclone, Beijing, China), compensated with insulin (5 mg/L, Sigma, USA), hydrocortisone (5 mg/L, Sigma, USA), penicillin/streptomycin (10 kU/L, Harbin Pharmaceutical Group, Harbin, China), epidermal growth factor (10 ng/mL, Invitrogen, Carlsbad, CA, USA) and FBS (10%). The cultured GMECs were evaluated by growth curve fitting, karyotype analysis, immunofluorescence staining (keratin, epithelial membrane antigen (EMA), vimentin, β-casein), oil red staining and RT-PCR of the β-casein gene and found that the methods we used in our group can obtain suitable GMECs with the function of secretion [46]. Prior to experiments, cells were changed to lipid-free, serum-free medium (basal medium plus 1 g/L bovine serum albumin; BSA) supplemented with prolactin (1.5 mg/L) to promote lactogenesis [48].

GMECs at 70%–80% confluence were transduced with adenovirus supernatant at a multiplicity of infection (MOI) of 200. Ad-*GFP* was used as the control. Cells were harvested for RNA extraction after transduction for 72 h using Cell RNA Extraction Kit (TIANGEN, Beijing, China).

### 4.7. Western Blot Analyses

The protein samples were extracted with radio immunoprecipitation assay lysis buffer (RIPA) (Solarbio, Beijing, China) supplemented with 10% PMSF. Western blot was assessed following the protocol described previously [49]. Polyclonal rabbit anti-SP1 (Abcam, ab13370, Cambridge, UK, 1:3000) and monoclonal mouse anti-GAPDH (CWBIO, China, 1:1000) were used as the primary antibodies. Polyclonal goat anti-mouse/rabbit IgG coupled to HRP (TIANGEN, AB101, China, and 1:1000) was used as secondary antibody respectively. Signals were detected using the chemiluminescent ECL Western blot detection system (Pierce, Thermo Fisher Scientific Inc., Rockford, IL, USA).

### 4.8. Statistical Analyses

The results are expressed as mean ± SD, and SPSS 19.0 (IBM, Armonk, NY, USA) was used for statistical analysis. Data of qPCR were analyzed relative to the control using the 2^−∆∆*C*t^ method. Group data for multiple comparisons were analyzed by ANOVA using a general linear model procedure followed by Tukey’s test. Significance was established at a *p* < 0.05.

## 5. Conclusions

In summary, we firstly cloned 4376 bp full-length cDNA of the *SP1* gene, includes 103 bp of 5'UTR, 2358 bp of ORF (HM_236311) and 1915 bp of 3'UTR using a RACE method. Goat *SP1* is predominantly expressed in the lungs and small intestine, followed by muscle, spleen, mammary gland and subcutaneous fat. Lactation stages have no significant effects on *SP1* expression. Over expression of *SP1* in GMECs led to higher concentration of *PPARγ* and lower *LXRα* mRNA levels in GMECs, and correspondingly altered the expression of *CD36*, *LPL*, *ACSL1*, *FABP3*, *ACACA* and *ACSS2*. These results were further enhanced by the silencing of *SP1*. The expression of *SREBP1* and FASN remained unaffected by the alteration of *SP1* mRNA. These results indicate that SP1 may be involved in milk fat metabolism via the control of PPARγ and LXRα.

## Supplementary Materials

Supplementary materials can be found at http://www.mdpi.com/1422-0067/16/01/1806/s1.
